# Sex and gender in depression after percutaneous coronary intervention: clinical patterns, mechanistic hypotheses, and care pathways

**DOI:** 10.3389/fpubh.2026.1952343

**Published:** 2026-10-07

**Authors:** Jieyi Wang, Zhiting Yang, Yang Hu, Zehua Liu, Hao Wu, Yilei Kong, Aisong Zhu

**Affiliations:** 1 Zhejiang Chinese Medical University, Hangzhou, Zhejiang, China; 2 Zhejiang Key Laboratory of Blood-Stasis-Toxin Syndrome, School of Basic Medical Sciences, Zhejiang Chinese Medical University, Hangzhou, China; 3 Zhejiang Engineering Research Center for “Preventive Treatment” Smart Health of Traditional Chinese Medicine, Zhejiang Chinese Medical University, Hangzhou, China

**Keywords:** cardiac rehabilitation, depression, gender, percutaneous coronary intervention, psychosocial risk, sex differences

## Abstract

Depressive symptoms are common after percutaneous coronary intervention (PCI) and are associated with poorer recovery, lower adherence to secondary prevention, reduced participation in cardiac rehabilitation, and a higher risk of adverse cardiovascular outcomes. Evidence also suggests that depression after PCI is not experienced, detected, or managed in the same way across sex and gender groups. Women are more often reported to have higher early symptom scores, greater anxiety comorbidity, more somatic symptom overlap, and lower access to some forms of guideline-based cardiovascular care. Men may be less likely to disclose affective symptoms and may present psychological distress through externalizing behaviors, treatment disengagement, smoking, alcohol use, anger, or delayed help-seeking. These patterns should not be interpreted as fixed male-female traits. Rather, they probably reflect interactions among biological sex-related factors, social gender norms, comorbidity, age, socioeconomic position, and healthcare access. Unlike previous reviews that pool depression across coronary syndromes, this review is organized around patients after PCI, explicitly distinguishes conclusions directly supported by post-PCI data from those extrapolated from broader coronary populations, and identifies the remaining extrapolations as research priorities. This narrative review summarizes clinical patterns of depression after PCI, discusses plausible biological and psychosocial mechanisms, and proposes a sex- and gender-informed care pathway for screening, referral, cardiac rehabilitation, and follow-up. The available literature supports a more cautious and individualized approach than a simple binary model: screening tools should be interpreted alongside clinical interview and behavioral indicators; rehabilitation should address practical barriers, stigma, caregiving burden, and functional goals; and future studies should report sex-disaggregated data while also measuring gender-related exposures.

## Graphical Abstract

**Figure:**
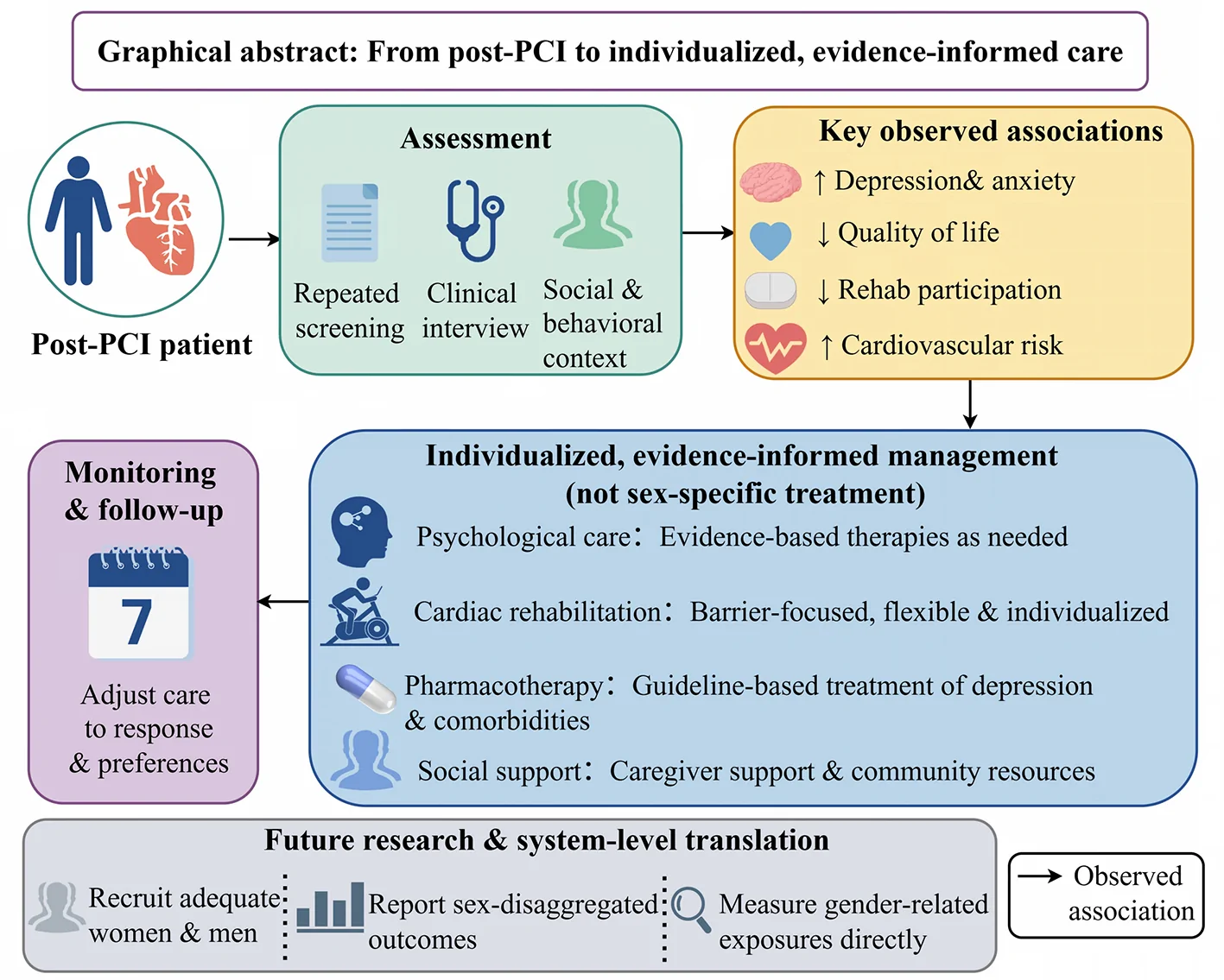
Depression after PCI should be assessed using repeated symptom screening linked to clinical interview, review of rehabilitation participation and adherence behavior, and assessment of social context. Findings should inform individualized referral, treatment, and follow-up rather than sex-based assumptions. PCI, percutaneous coronary intervention.

## 1 Introduction

Coronary artery disease has long been studied and treated through a male-centered clinical lens (1, 2). This history contributed to the under-recognition of cardiovascular symptoms in women, lower enrollment of women in cardiovascular trials, and delayed attention to sex-specific and gender-related care gaps (3, 4). The term “Yentl syndrome” remains useful as a reminder of these inequities, although contemporary research should avoid treating women as a homogeneous group or assuming that all disparities arise from biology alone (5).

Percutaneous coronary intervention (PCI) is now a routine revascularization strategy for acute and chronic coronary syndromes (6, 7). Recovery after PCI, however, is not limited to angiographic success or relief of ischemia (8, 9). Depression after PCI can interfere with medication adherence, lifestyle change, cardiac rehabilitation attendance, symptom interpretation, sleep, pain perception, and patient-clinician communication (10). These effects matter clinically because depression after coronary events has been linked to worse quality of life and adverse cardiovascular outcomes (11, 12).

Sex and gender may shape post-PCI depression through different but overlapping routes (5). Sex-related factors include ovarian and testicular hormone profiles, menopausal status, autonomic regulation, inflammation, platelet activity, microvascular dysfunction, and cardiometabolic comorbidity (13). Gender-related factors include caregiving roles, employment insecurity, illness stigma, norms about emotional disclosure, health literacy, social support, discrimination, and the way clinicians interpret symptoms (14). In practice, these domains interact (5). A woman with microvascular angina, caregiving burden, and delayed referral to rehabilitation may have a different risk profile from a man whose depressive symptoms are expressed through anger, smoking relapse, or non-adherence (15).

This article is a narrative review (5). It does not attempt a pooled estimate of prevalence or treatment effect. Instead, it synthesizes clinical and mechanistic literature relevant to depression after PCI, highlights where evidence is direct vs. extrapolated, and identifies practical points for sex- and gender-informed care (16).

Several recent reviews have examined depression in broader coronary populations, including depression after myocardial infarction (17), depressive symptoms in coronary heart disease (18, 19), psychological distress in cardiac rehabilitation (20, 21), and the prognostic impact of depression after PCI (22); previous literature has identified several predictors of post-PCI depression (16, 23). The present review differs from these earlier syntheses in three ways. First, it focuses specifically on patients who have undergone PCI and on sex- and gender-related patterns of post-PCI depression, a dimension that previous PCI-focused syntheses have addressed only in passing. Second, it makes the provenance of the evidence explicit: conclusions in Sections 4–7 are graded according to whether they are directly supported by post-PCI data or rely on extrapolated evidence from adjacent coronary populations, and the provenance of the key conclusions is summarized in Table 1. Third, it translates the available evidence into a practical, sex- and gender-informed care pathway spanning screening, referral, cardiac rehabilitation, and follow-up. In these respects, the present review is complementary to, rather than redundant with, earlier syntheses.

**Table 1:** Evidence provenance of key conclusions on depression after PCI.

Key conclusion	Main supporting population	Direct post-PCI evidence	Interpretation
Higher early depressive symptom scores in women after PCI	Post-PCI and revascularization cohorts (27, 30, 33, 36)	Partially supported	Supports higher early symptom burden in women; estimates vary by timing, instrument, and outcome definition
Depressive trajectories differ by assessment timing	Post-PCI cohorts (24, 28, 31, 38)	Partially supported	Timing of assessment materially affects reported trajectories
Sex differences in symptom expression	CHD and MI populations, male psychology literature (25, 39, 63)	Extrapolated	Candidate explanation; requires post-PCI confirmation
Socioeconomic and healthcare-access modifiers	General coronary populations (14, 45)	Extrapolated	Possible effect modifiers; largely unmeasured in post-PCI studies
Estrogen and gonadal hormone pathways	Basic science; CHD and MI populations (57, 59, 60)	Hypothesis only	Mechanistic hypothesis; no post-PCI confirmation
Autonomic dysfunction and heart rate variability	Post-MI revascularized cohorts (51)	Partially supported	Relevant to post-PCI depression but not directly tested in post-PCI cohorts
Inflammation, endothelial and microvascular mechanisms	CHD and MI populations (15, 47, 54, 55)	Extrapolated	Candidate pathways; no post-PCI confirmation
Masculinity, stigma and help-seeking behavior	General male psychology literature (39, 63)	Extrapolated	Candidate explanation; requires post-PCI testing
Healthcare-system disparities in referral and care	Coronary populations and post-PCI studies (65, 66, 88)	Partially supported	Modifiable service-level factors with partly direct evidence
Symptom case-finding linked to diagnostic evaluation	CHD/MI guidance, psycho-cardiology consensus, and post-PCI observational studies (24, 65, 68)	Partially supported	Screening should be linked to assessment, referral, and follow-up
Sex-adapted cardiac rehabilitation delivery	CHD and CR populations, including women-focused CR guidance (69, 70, 72, 74, 75)	Mostly extrapolated	Barrier-focused CR delivery supported; sex-specific post-PCI trials needed
SSRI use with dual antiplatelet therapy	Post-PCI cohort (76)	Directly supported	Directly supported by post-PCI data

Evidence categories indicate the degree to which each conclusion is supported by studies conducted specifically in post-PCI populations.

CHD, coronary heart disease; CR, cardiac rehabilitation; MI, myocardial infarction; PCI, percutaneous coronary intervention; SSRI, selective serotonin reuptake inhibitor.

## 2 Methods

We conducted a narrative review of the peer-reviewed literature on sex- and gender-related patterns of depression after PCI. Between inception and June 2026, structured searches were performed in PubMed/MEDLINE, Embase, PsycINFO, CINAHL, Web of Science, and the Cochrane Library. Search terms combined three domains: (i) mental health terms (depression, depressive symptoms, anxiety, psychological distress, mental health); (ii) revascularization terms (percutaneous coronary intervention, PCI, coronary angioplasty, coronary stenting, coronary revascularization); and (iii) sex and gender terms (sex, gender, sex differences, gender differences, women, men, female, male). A further search combined mental health terms with cardiac rehabilitation. Reference lists of retrieved reviews and key articles were screened for additional relevant publications, and guideline documents and consensus statements were consulted where applicable. We included peer-reviewed articles published in English or German that reported on adults and addressed depression or depressive symptoms in patients after PCI or in adjacent coronary populations from which evidence was extrapolated, including acute coronary syndrome, myocardial infarction, stable coronary disease, and cardiac rehabilitation. Eligible publication types comprised original studies (prospective and retrospective cohorts, cross-sectional studies, randomized trials), systematic reviews and meta-analyses, narrative reviews, and clinical guidelines or expert consensus statements. Evidence was classified according to its provenance. Direct post-PCI evidence was defined as findings obtained in cohorts or trials in which the study population consisted of patients who had undergone PCI, including sex- or gender-stratified analyses within post-PCI samples. Extrapolated evidence was defined as findings derived from adjacent populations that are relevant to post-PCI depression but have not yet been confirmed in post-PCI cohorts. Operational definitions of the clinical terms used are detailed in Section 3, and the provenance of the key conclusions is summarized in Table 1. Because this is a narrative review, the findings are presented as a structured synthesis rather than as pooled estimates, and no formal risk-of-bias assessment or meta-analysis was performed.

## 3 Scope of the review and interpretation of evidence

The review focuses on adults undergoing PCI and on studies addressing depressive symptoms, anxiety-depression overlap, quality of life, cardiac rehabilitation, and cardiovascular outcomes after coronary revascularization (16). Because the specific literature on sex and gender in post-PCI depression is still limited, some mechanistic discussion necessarily draws on adjacent evidence from acute coronary syndrome, myocardial infarction, stable coronary disease, depression biology, and cardiac rehabilitation (23). These extrapolations are labeled cautiously in the text and should be tested in prospective post-PCI cohorts (5).

Throughout the review, “sex” refers to biological attributes such as hormones, reproductive stage, chromosomes, body composition, inflammatory profiles, and vascular biology (5). “Gender” refers to socially patterned roles, behaviors, identities, expectations, and institutional practices (5). Many studies report only binary sex categories and do not directly measure gender-related variables; this limits causal interpretation and should be acknowledged in future research. Throughout this review, we use “depressive symptoms” to refer to scores obtained from validated self-report instruments, “screening positivity” to refer to scores above a predefined threshold, and “depression” or “clinically diagnosed depression” to refer to diagnoses established through clinical interviews or formal diagnostic criteria. We also distinguish pre-existing depression, defined as depression present before PCI, from new-onset post-PCI depression and transient procedural distress, as these represent clinically distinct outcomes with different implications for screening and management (24–26).

## 4 Epidemiological and clinical patterns

In several post-PCI studies, the prevalence and pattern of depressive symptoms varied by instrument, cutoff, and timing. In a Jordanian cross-sectional study of 335 patients with coronary artery disease assessed 10 days after elective PCI, 34.0% screened positive for moderate-to-severe depressive symptoms on the Patient Health Questionnaire-9 (PHQ-9; cutoff 10 or higher) and 45.1% for mild symptoms, and female sex, low educational level, and diabetes mellitus were independent predictors (27). In a Chinese prospective cohort of 610 PCI patients followed at 1, 3, and 6 months, anxiety and depressive symptoms were above the Hospital Anxiety and Depression Scale (HADS) threshold and depressive symptoms worsened over time (*p* < 0.001) (28). In another Chinese cross-sectional study of 202 patients after PCI, negative emotions were present in 63.8% of patients, with depression in 39.6% and anxiety in 8.4%, and female sex, age, educational level, income, and sleep status were associated with negative emotion scores (29). A 2025 study of older adults acute coronary syndrome patients treated with PCI found that Self-Rating Anxiety Scale (SAS) and Self-Rating Depression Scale (SDS) scores did not differ by sex before PCI, but were higher in women than in men at 1, 4, and 12 weeks after PCI; this adds recent direct post-PCI evidence but remains limited to an older single-center ACS cohort and symptom scales rather than diagnostic interviews (30). In the EXIT cohort of 638 exhausted PCI patients followed for 18 months, distinct courses of anxiety were identified and predicted subsequent outcomes (31). By contrast, a German study of patients 6 months after stent implantation found no significant sex difference in the prevalence of depressive symptoms, although sleep disorders were more common in women (32). In the DANAMI-2 trial of ST-elevation myocardial infarction, women had worse scores on several quality-of-life and psychological endpoints at 1 and 12 months, and the benefit of primary PCI relative to fibrinolysis differed by sex (33). Among US veterans undergoing PCI (*n* = 64,757; 1,040 women), adjusted sex differences in 1-year clinical outcomes were small (34); this study did not assess depressive symptoms. In a Norwegian cohort of 9,013 first-time PCI patients, anxiety and depression differed by cardiac rehabilitation participation at baseline, but participation was not associated with different 3-year symptom trajectories (35). These findings, summarized in Table 2, indicate that the evidence on sex differences in post-PCI depression is heterogeneous and depends substantially on study design, instrument, cutoff, timing, PCI indication, and whether the outcome is depressive symptoms, anxiety, quality of life, participation, or formally diagnosed depression.

**Table 2:** Evidence map of selected post-PCI and revascularization studies.

Study	Population and setting	Sample (women %)	PCI indication	Assessment (tool, timing)	Principal findings
Rawashdeh et al. (27)	CAD patients, Jordan	335 (24.8%)	Elective PCI	PHQ-9, 10 days post-PCI	34.0% moderate-to-severe depressive symptoms; female sex a predictor
Peng et al. (30)	Older adults ACS patients treated with PCI within 1 week, China	234 (48.3%)	ACS PCI	SAS and SDS, before PCI and 1/4/12 weeks post-PCI	Female patients had higher anxiety and depression symptom scores than male patients after PCI; single-center older adults ACS cohort and symptom-scale based
Liu et al. (28)	PCI patients, China	610 (18.2%)	Mixed	HADS, 1/3/6 months	Anxiety and depression above threshold; depression worsened over time
Yang et al. (29)	CHD patients post-PCI, China	202 (30.2%)	Mixed	IDA, cross-sectional	Negative emotions 63.8%; depression 39.6%; sex, income, sleep associated
Pedersen et al. (31)	Exhausted PCI patients, Netherlands (EXIT)	638 (22.0%)	Mixed	Anxiety scale, 18 months	Distinct anxiety courses predicted outcomes
Ladwig et al. (32)	Post-stent patients, Germany	335 (24.6%)	Stent implantation	Self-report, 6 months	No significant sex difference in depressive symptoms; sleep disorders more common in women
Olsen et al. (35)	First-time PCI patients, Norway	9,013 (21.8%)	First-time PCI	HADS, rehabilitation setting	Baseline anxiety and depression differed by rehabilitation participation; no differential 3-year symptom change
Farmer et al. (34)	US veterans, VHA	64,757 (1.6%)	Mixed PCI	Administrative data, 1 year	Sex differences in 1-year outcomes small after adjustment
Mortensen et al. (33)	STEMI, Denmark (DANAMI-2)	NR	Primary PCI vs fibrinolysis	SF-36, 1 and 12 months	Women worse on several endpoints; treatment benefit differed by sex
Frazier et al. (47)	Stent patients, US	490 (NR)	Elective stenting	Single-item screen, 4–12 h; 24-month outcomes	Depression screening and inflammatory proteins predicted MACE
Zhang et al. (22)	PCI cohorts (meta-analysis)	3,297 (NR)	Mixed PCI	Various instruments	Depression associated with adverse outcomes (RR 1.57, 95% CI 1.28–1.92)

NR, not reported; CHD, coronary heart disease; IDA, Irritability-Depression-Anxiety scale; MACE, major adverse cardiac events; RR, risk ratio; ACS, acute coronary syndrome; CAD, coronary artery disease; DANAMI-2, Danish Multicenter Randomized Study on Fibrinolytic Therapy vs. Acute Coronary Angioplasty in Acute Myocardial Infarction-2; EXIT, Exhaustion Intervention Trial; HADS, Hospital Anxiety and Depression Scale; PCI, percutaneous coronary intervention; PHQ-9, Patient Health Questionnaire-9; SAS, Self-Rating Anxiety Scale; SDS, Self-Rating Depression Scale; SF-36, 36-Item Short Form Health Survey; STEMI, ST-elevation myocardial infarction; VHA, Veterans Health Administration.

Several post-PCI and revascularization studies report higher depressive or anxiety-depression symptom scores among women, particularly during early recovery, but the signal is not uniform across cohorts (27, 30, 33, 36). This pattern is clinically plausible: women undergoing PCI are often older, have more comorbidities, may have a higher burden of non-obstructive coronary disease or microvascular dysfunction, and may face more practical barriers to rehabilitation (13). However, reported sex differences vary by population, indication for PCI, depression scale, baseline mental health status, and timing of assessment. It is therefore more accurate to describe the evidence as suggestive rather than definitive (16). Complementary evidence from a prospective community-based cohort of 7,735 adults in Southwest China showed that depression was associated with an increased risk of incident cardiovascular disease and acute myocardial infarction, while the associations of depression with cardiovascular disease and stroke appeared to be stronger among women. Because this study examined incident cardiovascular disease rather than depression after PCI, its findings should be interpreted as contextual rather than direct post-PCI evidence (37).

Timing is important (31, 32, 38). Some cohorts show higher early distress among women, with partial convergence over later follow-up (27, 32). Men may show lower screening positivity at early time points but later functional decline, behavioral disengagement, or delayed disclosure of symptoms (39). These observations argue for repeated assessment rather than a single discharge-period screen (24). Follow-up at 1 month, 3–6 months, and 12 months may capture different clinical phenotypes (28).

Measurement also matters (25, 40, 41). HADS and PHQ-9 are widely used and clinically useful, but they do not fully resolve sex- and gender-related differences in symptom expression (42). Women may be more likely to report sadness, anxiety, fatigue, sleep disturbance, appetite change, or bodily symptoms that overlap with cardiac recovery (39). Men may underreport affective symptoms or present distress through irritability, substance use, risk-taking, work withdrawal, or poor adherence (25). These patterns do not invalidate standard tools, but they support combining questionnaires with interview-based assessment, behavioral review, and collateral clinical information when risk is suspected.

In addition, the comparability of studies is limited by differences in instruments, cutoffs, and timing of assessment relative to PCI. Reported prevalence estimates range widely depending on whether the HADS, PHQ-9, Beck Depression Inventory (BDI), or Center for Epidemiologic Studies Depression Scale (CES-D) is used, on the chosen cutoff, and on whether symptoms were present before PCI or developed during recovery. For example, using the PHQ-9 with a cutoff of 10 or higher, 34.0% of patients screened positive 10 days after elective PCI in one cohort (27), whereas prevalence estimates based on other instruments and time points differ considerably (28, 31, 35). Transient procedural distress, positive screening scores, and clinically diagnosed depression represent distinct clinical entities with different implications for management; they should not be conflated, and future studies should report them separately (24–26).

The influence of timing is illustrated by a prospective study of 79 patients treated with primary PCI for ST-elevation myocardial infarction, in which the proportion of patients with depressive symptoms on the BDI-II fell from 21.5% within 24 h of the procedure to 9.2% before discharge, then rose again to 15.4% at 6 months and 13.8% at 12 months (43). Similarly, in survivors of myocardial infarction, reported depression prevalence differs markedly depending on the instrument and cutoff used, and screening followed by structured clinical interview, with reassessment several weeks after discharge, has been recommended (44).

Depression after PCI should not be separated from social position (14). Income, education, employment, family responsibility, transportation, insurance, social support, race or ethnicity, discrimination, and local access to rehabilitation can all modify risk (14, 45). Current post-PCI studies rarely measure these variables with enough detail (14). As a result, some apparent sex differences may partly reflect unmeasured socioeconomic and healthcare-access differences (5).

## 5 Sex-related biological pathways

The mechanisms discussed in this section are classified according to the provenance and directness of their supporting evidence: (1) mechanisms examined in post-PCI cohorts; (2) mechanisms supported by evidence from broader coronary disease populations; and (3) mechanisms that are biologically plausible but have not yet been tested in the post-PCI setting. This distinction is important because several of the mechanisms below represent well-established general sex differences rather than mechanisms demonstrated to explain sex differences in post-PCI depression; they should therefore be regarded as candidate explanations pending direct testing.

This cautious interpretation is consistent with the broader literature on depression and coronary heart disease, in which no single biological or behavioral mechanism accounts for more than a small proportion of the risk (18).

In addition, several mechanisms discussed below, such as the effects of gonadal hormones on mood and neuroplasticity, represent well-established general sex differences in affective disorders rather than mechanisms shown to explain sex differences in post-PCI depression specifically (46).

Evidence category 1: directly examined in post-PCI cohorts. Direct post-PCI evidence is available for biological correlates and symptom-outcome pathways, although no study has yet established a validated sex-specific mechanism. In 490 patients undergoing successful elective coronary stenting, Frazier et al. measured inflammatory proteins 4–12 h after the intervention and found that positive depression screening was more common in women and was associated in unadjusted analyses with several inflammatory proteins; both depression screening and selected inflammatory markers were also associated with subsequent major adverse coronary events (47). In a prospective study of 796 PCI patients, PHQ-2 symptoms assessed after PCI were associated with death or non-fatal myocardial infarction, with a stronger association in men than in women, although the sex interaction did not reach conventional statistical significance (48). A prospective cohort of 150 patients with acute coronary syndrome reported that early post-stenting depressive symptoms, assessed 48–72 h after the procedure, were associated with baseline depressive symptoms, post-operative anxiety, and recent major life events (49). In a 2025 AMI-PCI cohort, monocyte count, C-reactive protein, albumin, Killip class, and left ventricular ejection fraction were associated with depressive symptoms 1 month after PCI (50). These findings support directly observed links among post-PCI symptoms, inflammatory or clinical burden, and outcomes, but they do not demonstrate mediation, causality, or a sex-specific biological pathway.

Evidence category 2: supported by data from post-myocardial infarction and revascularized cohorts, but not yet confirmed in post-PCI depression studies. Autonomic dysfunction is another plausible bridge between cardiovascular injury and depression (51, 52). Reduced heart rate variability after myocardial infarction and revascularization has been linked to prognosis, stress physiology, and emotional regulation (51). Whether women have a more pronounced or clinically meaningful autonomic vulnerability after PCI requires more direct study (51). In future cohorts, HRV, sleep, physical activity, and inflammatory markers should be interpreted together rather than as isolated signals (51, 53).

Evidence category 2: supported by data from post-myocardial infarction and revascularized cohorts, but not yet confirmed in post-PCI depression studies. Inflammation, endothelial dysfunction, and microvascular disease may help explain why some patients continue to feel fatigued, dyspneic, or functionally limited despite technically successful PCI (15, 54). Women have a higher prevalence of some non-obstructive and microvascular coronary phenotypes, which can complicate symptom interpretation (15, 55). Gender-specific associations between matrix metalloproteinases and inflammatory markers—including an “IL-6/MMP-3” pattern in women and a “CRP/MMP-9” pattern in men—have been described in post-myocardial infarction patients (56). Whether these patterns constitute distinct sex-specific axes in post-PCI depression remains to be established, and they are best regarded as candidate pathways for future validation in post-PCI cohorts (47).

Evidence category 3: biologically plausible, but not tested in post-PCI cohorts. Several biological pathways may contribute to differences in mood symptoms after PCI, although few have been tested directly in sex-stratified post-PCI depression cohorts (57, 58). Estrogen signaling has plausible relevance because estradiol affects serotonergic transmission, dopaminergic modulation, neuroplasticity, immune activity, endothelial function, and autonomic regulation (57, 59, 60). Perimenopause and post**-**menopause may therefore be periods of increased vulnerability for some women (57). This does not mean that estrogen withdrawal alone explains post-PCI depression; age, comorbidity, sleep, pain, inflammation, and psychosocial stress are likely to contribute (23).

Evidence category 3: biologically plausible, but not tested in post-PCI cohorts. Testosterone decline in older men has also been associated with mood, motivation, fatigue, and metabolic health, but its role in post-PCI depression remains uncertain (61, 62). Low testosterone should not be used as a stand-alone explanation for depressive symptoms, and hormone-based interventions cannot be inferred from the current PCI literature (61).

## 6 Gender-related psychosocial and healthcare pathways

In this section we distinguish observed sex-related findings from gender-related hypotheses. The sex-related findings reviewed above, such as higher depressive symptom scores among women in several post-PCI cohorts, were measured directly in the included studies. By contrast, the gender-related explanations discussed below, including masculinity norms, caregiving burden, stigma, and emotional disclosure, were rarely measured as exposures in the post-PCI samples included in this review. They are therefore presented as hypotheses that require explicit measurement of gender-related variables in future post-PCI cohorts, rather than as established explanations of the observed sex differences.

Gender norms can shape how patients interpret symptoms and whether they ask for help (63). Men who strongly endorse ideals of self-reliance or emotional control may describe distress as fatigue, irritability, loss of productivity, or frustration rather than sadness (63). Some may delay psychological care or disengage from medication and rehabilitation (39). Women may face different pressures, including caregiving demands, lower income, lower referral to rehabilitation in some settings, or having cardiac symptoms attributed to anxiety or non-cardiac causes (64).

Healthcare systems can amplify these differences (65). Delayed recognition of cardiovascular symptoms in women, lower rates of referral or participation in rehabilitation, under-treatment of psychiatric comorbidity, and fragmented cardiology-mental health pathways may increase distress after PCI (65, 66). These are modifiable service problems, not inherent patient characteristics.

Discrimination and chronic stress deserve more explicit attention (67). Evidence from coronary disease populations suggests that discrimination may relate to biological aging, stress physiology, and inflammatory burden (67). For post-PCI depression, this evidence remains indirect but relevant (67). Future studies should measure discrimination, social isolation, household burden, employment stress, and perceived stigma alongside conventional cardiovascular variables (14) (Figure 1).

**Figure 1:**
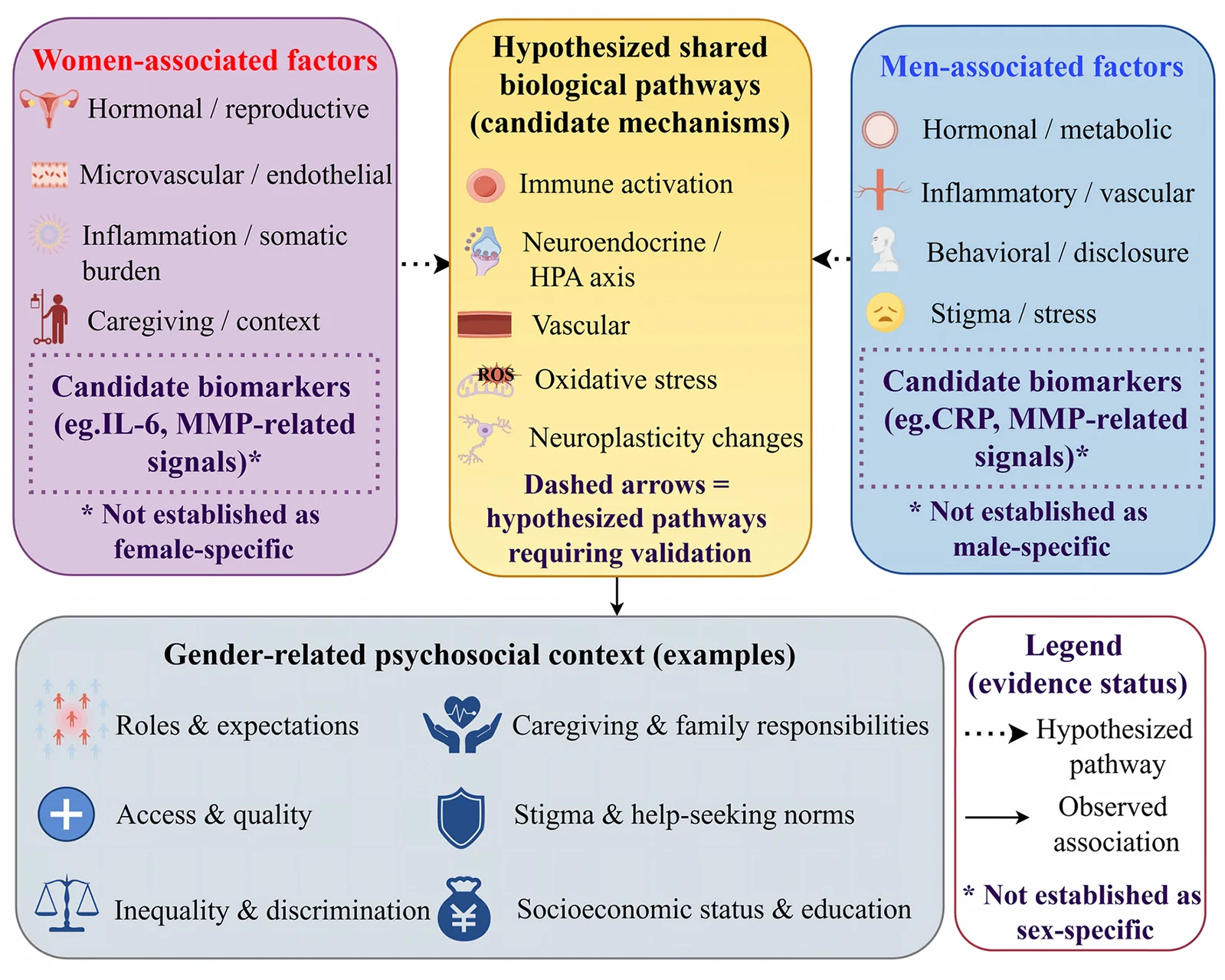
Proposed sex- and gender-informed model of depression after PCI. Biological sex-related factors, gender-related contexts, clinical presentation, and healthcare-system factors interact to influence depressive symptoms, recognition, treatment engagement, and cardiovascular recovery after PCI. Arrows indicate reciprocal and potentially modifying relationships rather than established causal pathways. PCI, percutaneous coronary intervention.

## 7 Sex- and gender-informed clinical care

To avoid overstating the clinical recommendations, this section distinguishes three levels of guidance. Established guideline-based care refers to case-finding, clinical assessment, referral, and follow-up practices recommended for coronary heart disease populations when a system for timely diagnostic evaluation and treatment is available. A positive screening score identifies symptoms that require clinical assessment; it does not by itself establish a depressive disorder. Extrapolated recommendations are derived from intervention studies in broader cardiovascular populations and have not been tested specifically in post-PCI cohorts. Proposed strategies are suggested by the authors on the basis of the mechanistic and psychosocial evidence reviewed above; they are directions for clinical consideration and future research rather than evidence-based standards. The review does not imply that women and men require predefined separate care pathways; care should be tailored to individual biological, psychological, and social circumstances.

A practical care model should begin before discharge. In settings with capacity for diagnostic evaluation, referral, treatment, and follow-up, clinicians can use brief symptom case-finding, review prior mental health history, assess suicide risk when indicated, and provide clear referral options. This conditional framing is consistent with contemporary psycho-cardiology guidance, including the 2025 ESC consensus statement, which emphasizes validated tools, integrated cardiovascular and mental-health care, stepped management, and follow-up rather than isolated screening (68). Screening should be repeated when clinically indicated because early post-procedural distress, persistent symptoms, and delayed-onset depression may represent different clinical trajectories.

For women, individualized assessment can consider anxiety-depression overlap, somatic symptom burden, residual ischaemic symptoms, caregiving responsibilities, transportation, income constraints, and social support. Family involvement may help some patients, but it should be offered as an option rather than assumed to be appropriate for all women. These are individualized assessment prompts, not sex-specific prescribing rules.

For men, individualized assessment can also allow for presentations that are less verbally emotional, including irritability, sleep disturbance, alcohol or tobacco use, work functioning, medication-taking, and withdrawal from usual activities. Goal-oriented rehabilitation may improve engagement for some patients, but psychological distress should not be hidden behind performance targets. These considerations are hypotheses and clinical prompts rather than validated male-specific post-PCI pathways.

Cardiac rehabilitation should be adapted to barriers rather than assigned by sex alone (69–71). Flexible scheduling, home-based or hybrid rehabilitation, motivational interviewing, cognitive behavioral strategies, peer support, smoking cessation, medication-adherence support, and return-to-work counseling can be combined according to individual need (72, 73). High-intensity interval training may be appropriate for selected stable patients after clinical risk stratification, but it should not be presented as a sex-specific default (74).

Evidence from broader cardiac rehabilitation settings suggests that automatic referral, a strong clinician recommendation, barrier-focused enrollment support, and integration of mental-health and social-support needs may improve participation among women (21, 75). These strategies are supported by women-focused cardiac rehabilitation literature and recent AHA scientific-statement guidance, but they have not been established as sex-specific depression interventions in post-PCI populations and should be adapted to local workflow and individual patient needs.

Pharmacological treatment of depression after PCI requires coordination between cardiology and mental health clinicians (76–78). Selective serotonin reuptake inhibitors are often considered when medication is indicated, but clinicians should evaluate bleeding risk, antiplatelet therapy, QT interval, drug interactions, age, renal function, and suicide risk (79–81). Psychotherapy, exercise-based rehabilitation, sleep treatment, and social support interventions should be integrated rather than treated as secondary additions (74, 82).

Intervention evidence specific to post-PCI populations remains limited. A 2025 Cochrane systematic review and meta-analysis of psychological interventions in coronary heart disease, heart failure, or atrial fibrillation found reductions in depression and anxiety and improvement in mental health-related quality of life, but no clear reduction in all-cause mortality or major adverse cardiovascular events; this strengthens the general cardiac evidence base but remains extrapolated rather than PCI-specific (83). Earlier randomized trials and systematic reviews in broader coronary populations also support psychological interventions, exercise-based cardiac rehabilitation, and home-based or hybrid rehabilitation delivery for psychological or quality-of-life outcomes (74, 80). The ENRICHD trial improved depression and social support after myocardial infarction but did not reduce cardiac events (82). Few trials were conducted specifically in post-PCI cohorts, and even fewer reported sex-stratified outcomes. Whether treatment efficacy, participation, adherence, or barriers to intervention differ by sex or gender in post-PCI settings remains uncertain and is an important evidence gap for future trials (Table 3).

**Table 3:** Intervention studies relevant to depression after PCI.

Study	Population	Design	Intervention	Main outcomes	Post-PCI or extrapolated
Appels et al. (EXIT) (89)	Exhausted angioplasty and PCI patients (*n* = 710)	RCT	Behavioral group intervention	Improved exhaustion and quality of life; effect on depression limited	Post-PCI population; no sex-stratified outcomes reported
Ski et al. (83)	CHD, heart failure, or atrial fibrillation patients	Cochrane systematic review and meta-analysis	Psychological interventions	Reduced depression and anxiety and improved mental health-related quality of life; no clear reduction in all-cause mortality or MACE	Broad cardiovascular population (extrapolated); not post-PCI-specific and sex-stratified evidence limited
ENRICHD Investigators (82)	Post-myocardial infarction depressed patients (*n* = 2,481)	RCT	Cognitive-behavioral therapy plus or minus SSRI	Improved depression and social support; no reduction in cardiac events	MI population (extrapolated); sex-stratified secondary analyses only
Richards et al. (85)	Coronary heart disease patients	Cochrane systematic review	Psychological interventions	Small reduction in depressive symptoms	Broad CHD population (extrapolated); sex-stratified data limited
Dibben et al. (74)	Coronary heart disease patients	Cochrane systematic review	Exercise-based cardiac rehabilitation	Improved quality of life; may reduce depressive symptoms	Broad CHD population including PCI (extrapolated); sex-stratified data limited
Anderson et al. (72)	Coronary heart disease patients	Cochrane systematic review	Home-based vs. center-based cardiac rehabilitation	Comparable clinical and psychosocial outcomes	Broad CHD population (extrapolated); no sex-specific analysis
Olsen et al. (35)	First-time PCI patients (*n* = 9,013)	Prospective cohort	Cardiac rehabilitation participation	Baseline anxiety and depression differed by participation; no differential 3-year symptom change	Post-PCI population; no sex-stratified intervention effect

CHD, coronary heart disease; SSRI, selective serotonin reuptake inhibitor; RCT, randomized controlled trial; MACE, major adverse cardiovascular events; MI, myocardial infarction; PCI, percutaneous coronary intervention.

Table 4 summarizes the major sex- and gender-informed risk domains that should be considered when assessing depression after PCI.

**Table 4:** Sex- and gender-informed risk framework for depression after PCI.

Domain	Sex-related observations	Gender-related context	Clinical implication	Caution
Hormones and reproductive stage	Estradiol and testosterone may influence mood, vascular function, inflammation, and autonomic regulation.	Symptoms may be interpreted differently across age, menopause status, and social expectations.	Record age, menopause status, hormone therapy, and relevant endocrine comorbidity when available.	Do not infer hormone treatment from association studies.
Coronary phenotype	Women may have more non-obstructive or microvascular coronary disease in some PCI-related populations.	Persistent symptoms may be dismissed as anxiety, which can delay appropriate care.	Assess residual ischemia, microvascular symptoms, and depression together.	Avoid assuming all somatic symptoms are psychiatric.
Inflammation/ autonomic function	Inflammation and HRV are plausible links between coronary events and depression.	Stress, sleep, discrimination, and caregiving burden may alter these pathways.	Consider repeated symptom assessment and objective recovery indicators in research cohorts.	Biomarker axes are not ready for routine sex-specific algorithms.
Symptom expression	Women often report higher anxiety-depression and somatic symptom burden in several studies.	Men may express distress through irritability, substance use, withdrawal, or non-adherence.	Use questionnaires plus interview and behavioral review.	Screening differences are not proof of true prevalence differences.
Rehabilitation access	Functional recovery may differ by age, comorbidity, and baseline exercise capacity.	Caregiving, work, transport, cost, stigma, and clinician referral patterns influence participation.	Offer flexible, hybrid, and barrier-focused CR options.	Do not assign CR format by binary sex alone.
Treatment planning	Drug response and adverse effects may vary by age, comorbidity, and concurrent antiplatelet therapy.	Preference, stigma, family support, and health literacy affect uptake.	Coordinate cardiology, primary care, rehabilitation, and mental health referral.	Monitor bleeding risk, QT interval, interactions, and suicide risk when medication is used.

CR, cardiac rehabilitation; HRV, heart rate variability; PCI, percutaneous coronary intervention; QT, QT interval.

## 8 Research priorities

Future studies should recruit sufficient numbers of women and men, report sex-disaggregated outcomes, measure gender-related exposures directly, and specify whether depression was present before PCI or newly developed afterward (5, 29, 34). Studies should also separate acute coronary syndrome from stable coronary disease, define PCI indication, and report follow-up time points consistently (16).

A stronger evidence base would include prospective cohorts with repeated depression screening, structured psychiatric assessment, cardiac rehabilitation data, medication adherence, biomarkers, autonomic measures, and patient-reported outcomes (51, 84). Randomized trials of sex- and gender-informed rehabilitation or psychological care should test predefined outcomes rather than relying on *post hoc* subgroup claims (85, 86).

Research should also move beyond a simple male-female binary when data allow (5). Gender identity, menopause status, hormone therapy, socioeconomic position, race or ethnicity, sexual orientation, caregiving burden, and healthcare access may all modify risk and treatment response (5, 87).

## 9 Limitations

Several limitations should be considered when interpreting this review. First, the number of studies that specifically examined sex- or gender-related patterns of depression in post-PCI cohorts remains limited, and several studies included in the epidemiological section were not designed for hypothesis-driven testing of sex and gender differences. Second, depression assessment varied across studies in terms of instrument (e.g., HADS, PHQ-9, BDI, CES-D), cutoff, and timing relative to PCI, which limits direct comparability of prevalence estimates and effect sizes; we therefore did not pool estimates. Third, a substantial proportion of the mechanistic and psychosocial evidence is extrapolated from adjacent populations, including acute coronary syndrome, myocardial infarction, stable coronary disease, cardiac rehabilitation participants, and general depression biology; these extrapolations are labeled throughout the text and summarized in Table 1, but they have not been confirmed in post-PCI cohorts. Fourth, most included studies reported binary sex categories and did not measure gender-related variables directly, which limits causal interpretation of gender-related mechanisms. Fifth, as a narrative review, study selection and synthesis involved author judgment, and no formal risk-of-bias assessment or quantitative synthesis was performed. Finally, publication bias in favor of positive or significant sex differences cannot be excluded.

## 10 Conclusion

To provide a balanced interpretation, the conclusions of this review are graded according to the strength of the underlying evidence. The evidence is relatively consistent that depressive symptoms are common after PCI and that several post-PCI cohorts report higher early symptom scores in women, although findings vary by timing and instrument. Evidence is more limited and partly conflicting for sex differences in symptom trajectories, treatment response, and rehabilitation outcomes. Biological and psychosocial mechanisms proposed to explain these differences, including hormonal pathways, autonomic dysfunction, inflammation, masculinity norms, and caregiving burden, remain largely hypothesis-generating because most were not tested directly in post-PCI cohorts or were extrapolated from broader coronary populations.

Available evidence suggests that sex-related factors and gender-related contexts may shape symptom patterns, detection, and recovery barriers after PCI, but direct post-PCI evidence remains limited and heterogeneous. The strongest clinical message is not that women and men require separate pathways, but that post-PCI care should be attentive to symptom phenotype, biological vulnerability, social context, rehabilitation access, and follow-up timing. A sex- and gender-informed approach can make assessment and rehabilitation more precise when it remains individualized, evidence-calibrated, and linked to appropriate clinical evaluation.
